# Multilevel hybrid method for optimal buffer sizing and inspection stations positioning

**DOI:** 10.1186/s40064-016-3756-2

**Published:** 2016-11-30

**Authors:** Fatima Zahra Mhada, Mohamed Ouzineb, Robert Pellerin, Issmail El Hallaoui

**Affiliations:** 1 GERAD and University Mohammed V-Rabat/ENSIAS, B.P. 713, Rabat-Instituts, Madinat Al Irfane, Rabat, Morocco; 2 GERAD and Institut National de Statistique et d’Economie Appliquée, B.P. 6217, Rabat-Instituts, Madinat Al Irfane, Rabat, Morocco; 3 CIRRELT and Polytechnique Montreal, CP 6079, succ. Centre-Ville, Montreal, QC Canada; 4 GERAD and Polytechnique Montreal, CP 6079, succ. Centre-Ville, Montreal, QC Canada

**Keywords:** Production, Inspection, Quality, Combinatorial optimization, Metaheuristics

## Abstract

Designing competitive manufacturing systems with high levels of productivity and quality at a reasonable cost is a complex task. Decision makers must face numerous decision variables which involve multiple and iterative analysis of the estimated cost, quality and productivity of each design alternative. This paper adresses this issue by providing a fast algorithm for solving the buffer sizing and inspection positioning problem of large production lines by combining heuristic and exact algorithms. We develop a multilevel hybrid search method combining a genetic algorithm and tabu search to identify promising locations for the inspection stations and an exact method that optimizes rapidly (in polynomial time) the buffers’ sizes for each location. Our method gives valuable insights into the problem, and its solution time is a small fraction of that required by the exact method on production lines with 10–30 machines.

## Background

The system we consider in this paper consists of *n* machines, *n* fixed-size buffers, and $m+1$ inspection stations (one inspection station is at the end of the line and *m* are internal) in series. We explore the impact of quality constraints on the system performance and (numerical) complexity. The objective is to simultaneously minimize the combined storage and shortage costs, determine the optimal buffers’ sizes, and specify the optimal number and positions of the *m* internal inspection stations. The resulting mathematical model is an intractable combinatorial nonlinear optimization model; it is difficult to find an exact solution in a reasonable time, especially when the production line is large. This paper develops an efficient evolutionary heuristic for this complex model. While the number of machines considered in the literature does not exceed 10 (to the best of our knowledge), this paper aims at solving larger production lines and a variable number of inspection stations.

In the recent past, several authors have investigated improving productivity with production control policies. Most studies assume that all the parts produced are conforming, which is unrealistic. Standard quality analysis models usually separate the problem of quality preservation in production lines (via the positioning of inspection stations, for example) and the optimization of production (via Kanban, CONWIP etc.). However, these two problems are interdependent (see Kim and Gershwin 2005, 2008; Colledani and Tolio 2005, 2006, 2009, 2011). We illustrate this by considering the Kanban strategies introduced by Toyota in the 1960s that have since become the paradigm of “lean manufacturing”. These strategies essentially advocate areas of limited storage between successive machines in a production line. The storage areas allow a degree of decoupling between machines to increase the productivity of the line. The idea is to limit the impact of shortages on downstream machines and to avoid blocking machines that are upstream from one that is broken down. These areas should be limited because they are associated with frozen capital: they introduce storage costs and extended transit times for parts in the workshop.

In an ideal “just in time” scenario, there would be no intermediate storage and finished parts would be pulled from the system as they are produced. In reality, machine breakdowns and shortages of raw material or operators make it impossible to ensure continuous workshop output. The sizes of the storage areas should depend on the likelihood of such events and the estimated costs of the associated service interruptions. If we decide the amount of storage without considering the quality dimension, we risk creating storage areas that contain important quantities of defective parts. These defective parts correspond to misused production-line time, and they undermine the effectiveness of the intermediate storage in terms of increasing productivity. Moreover, the costs associated with storage impact the budget available for improvements in quality and vice versa. In a similar manner, Inman et al. (2013) show that production design impacts quality and vice versa. Also, the location of an inspection station affects both the expected production cost per item and the production rate of the line. The quality control is obviously critical to any production system and has significant managerial impact. As a result, it is crucial to develop strategies to allocate inspection or quality control stations into a manufacturing processes that can help preventing all wastes resulting from unidentified defective items being processed (see Shetwan et al. 2011; Raviv 2013).

In a real manufacturing context, simulation can be used to determine the optimal buffers’ sizes and the optimal positions of the inspection stations by considering all possible scenarios. However, this is not practical when the systems are complex: the number of scenarios is $(^n_m) \times 100^n$ for a system with *n* machines and *m* inspection stations where each buffer of equal size is discretized to 100 levels. Note that the system is required to have an inspection station at the end of the line. Since the position of this station is known, it is not included in the optimization. An alternative to simulation is to develop a realistic model and a fast optimization technique to solve it. The real model is however very complex because many factors impact quality and production. So, we need elaborate some simplifying assumptions in such a way that the resulting model is still realistic and can be solved optimally.

Researchers have investigated the integration of quality aspects and production policies for a single unreliable machine producing a single product type. The system starts in an “in-control” state producing conforming items and then switches to an “out-of-control” state and starts producing nonconforming items. Integrated models can be classified as: (1) integrated production and quality management (e.g., Hajji et al. 2010; Kutzner and Kiesmüller 2013; Mhada et al. 2014b; Naebulharam and Zhang 2014; Matta and Simone 2016; 2) integrated production and maintenance management (e.g., Ben-Daya 2002; Dhouib et al. 2012; Rivera-Gómez et al. 2013; Jafari and Makis 2015; Nourelfath et al. 2016); and (3) integrated production, quality, and maintenance management (e.g., Radhoui et al. 2009, 2010; Njike et al. 2012; Zequeira et al. 2008; Rivera-Gmez et al. 2016; Bouslah et al. 2016). Some researchers (see Mandroli et al. 2006) focus on determining the optimal inspection-station position in *n* serial production lines with or without (1) scrapping, (2) reworking, and (3) offline repairs, without considering the buffer sizing.

A large part of published articles related to the inspection suppose the inspection reliable and without errors. They assumed that the produced items are subject to 100% inspection and there are no inspection errors. Some researchers have incorporated quality inspection errors caused, among others, by human failure in their models (see Hsu and Hsu 2013; Duffuaa and Khan 2002; Khan et al. 2014).

In the context of serial production lines with production and inspection machines that follow Bernoulli reliability and quality assumptions, Meerkov and Zhang (2010, 2011) provide important insight into the nature of production and quality bottlenecks. Such systems are encountered in automotive assembly and painting operations where the downtime is relatively short and the defects are a result of uncorrelated random events (Ju et al. 2013).

Mhada et al. (2014a) consider a situation where every machine has to satisfy a demand specified as good parts per time unit. Moreover, the inventory size takes into account that the stock is a mixture of good and defective parts, and defective parts are generally eliminated at inspection stations. Mhada et al. (2014a) improve the continuous models of Kim and Gershwin (2005, 2008). The former model is more fluid in the sense that quality is treated as a continuous flow, whereas in the Kim and Gershwin models quality is considered to be discrete. They considered a line with 4 machines.

Mhada et al. (2014a) develop decomposition methods to reduce the analysis of the line to a series on an equivalent machine that can be isolated and sequentially analyzed. They consider just one inspection station. Ouzineb et al. (2013, 2014) generalize the model proposed in Mhada et al. (2014a) by optimizing the number of internal inspection stations ($m \ge 1$) and their positioning, and develop an exact search method to solve it. The exact search method (ESM) is exhaustive: the search is guaranteed to generate all possible locations of inspection stations. For each location, the problem is reformulated as a network flow optimization problem that can be efficiently solved by a fast polynomial algorithm. This method is efficient for small instances, but it may need days for reasonably large instances. The solution time increases exponentially with the size of the problem, i.e. with *n* and *m*. For larger problems, we expect that the solution time may span over several months. We can have an idea on that from Table 4.

The present paper efficiently adapts a space partitioning technique that decomposes the search space into multiple levels. At each level, we use two well-known metaheuristics to explore some potential regions. A genetic algorithm (GA) is used to identify a population of promising locations of inspection stations. Tabu search (TS) then searches intensively around by exploring locations that are neighbors to some newborns in the population. The population evolves dynamically in a sense that worst locations are discarded and good ones are added during the search. We use a quick shortest path procedure (from ESM) to determine the optimal buffers’ sizes and evaluate the objective function (the fitness) for each considered location. From time to time, GA replenishes the population. GA facilitates thus the exploration by guiding the search to unvisited regions with good potential, and this gives a certain diversification in terms of the regions to explore. The new approach is hence a multilevel hybrid heuristic (MHH) that provides certain balance between diversification and intensification. This hybrid approach is effective when the number of solutions is huge: it finds optimal or near-optimal solutions in a fraction of the time required by ESM. We also discuss some important properties of the production line and its sensitivity to system parameters.

Similar ideas have been used to solve some high-dimensional combinatorial optimization problems in other contexts. In Ouzineb et al. (2011), the authors apply a GA to solve an optimal design model for assembly/disassembly manufacturing networks. The objective is to maximize the production rate subject to a total cost constraint, the machines are chosen from a list, and the buffers’ sizes are within a predetermined range. Nahas et al. (2014) applies a GA–TS algorithm to optimize the nonhomogeneous redundancy of multistate series-parallel systems. In Ouzineb et al. (2010), the authors use space partitioning to solve two design optimization problems: the first is the expansion scheduling of multistate series-parallel systems, and the second is the redundancy allocation of binary-state series-parallel systems. We adapt space partitioning and GA–TS techniques to efficiently solve the design problem addressed in this paper.

The remainder of this paper is organized as follows. “Problem formulation and discussion” section presents the problem statement and discusses some theoretical properties, and “Methods” section describes our approach. “Numerical results” section presents numerical results for ESM (Ouzineb et al. 2013, 2014) and our method. “Conclusions and future work” section provides concluding remarks.

## Problem formulation and discussion

**Fig. 1:**
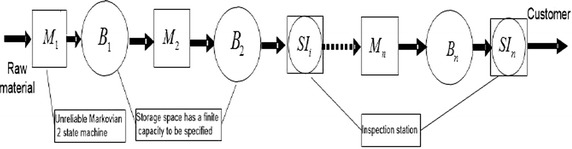
Production line

Figure 1 illustrates the production system studied. It consists of *n* machines separated by *n* buffers with $m+1$ inspection stations. The machines can be either up or down, starved or blocked. Machine $M_i$ is starved if one of the upstream machines is down and all the buffers between that machine and $M_i$ are empty. $M_i$ is blocked if one of the downstream machines is down and all the buffers between that machine and $M_i$ are full. When an operating machine is neither starved nor blocked, it continuously transfers parts from the upstream buffer to the downstream machines. We assume that the first machine can never be starved, and an inspection station is located after the last machine to ensure the quality of the parts received by the customer. The machines $M_i, i=1 \ldots n$ can be modeled as a continuous-time Markov chain that produces a single part type with two quality levels, conforming and nonconforming, with a predefined ratio $\beta$ of nonconforming parts to conforming parts. We assume that the machines have the same maximum production rate *k*, failure rate *p*, and repair rate *r*.

Let *d* be the demand rate for conforming parts, $x_i$ the inventory level for buffer *i*, $\tilde{d}_i$ the long-term average number of parts pulled per unit of time from stock $x_i$, $c_p$ the storage cost per time unit and per part, $c_I$ the inspection cost per pulled part, and $a^n_{des}$ the required availability rate of conforming finished parts.

A binary variable $\lambda _i$ determines whether or not there is a station before machine $M_{(i+1)}$ to identify nonconforming parts. We assume that there are *m* inspection stations in the line (*m* is unknown), i.e., $\sum \nolimits _{i=1}^{n-1} \lambda _i =m$, $m \in \{0,1, \ldots , (n-1)\}$ and $\lambda _n =1$. The problem is to minimize the long-term average global cost per unit time of storage, shortages, and inspection:

1 $$\begin{aligned} {J_T}_{(\tilde{d}_i, \lambda _i) } = \lim _{T \rightarrow \infty } \frac{1}{T}\,\sum _{i=1}^{n} E \left[ \int \limits _0^T ( c_p\, x_i(t) + \,c_I \,\tilde{d}_i\,\lambda _i) dt \right] . \end{aligned}$$

The constraints are $\sum _{i=1}^{n-1} \lambda _i =m$, $\lambda _i$ binary ($\lambda _n =1$), and $m \in \{0,1, \ldots , (n-1)\}$. The objective function (1) can be rewritten as (see Mhada et al. 2014a):

2 $$\begin{aligned} J(a, \lambda ) = \sum _{i=1}^{n-1} T^{(i)}( a_i, a_{i-1},\lambda ) + T_F(a^n_{des}, a_{n-1},\lambda )+ c_I\,\sum _{i=1}^{n} \lambda _i \, \tilde{d}_i \end{aligned}$$

with $a=(a_1, a_2, \ldots , a_{n-1})$ and $\lambda = ( \lambda _1, \lambda _2, \ldots , \lambda _{n-1})$. Here,

- $a_i, i=1 \ldots n-1$ is the total work-in-progress availability coefficient at buffer $x_i$; this coefficient has a lower bound of $max\left[ \left( \frac{r}{(r+p)}\right) ^{i-1},\frac{(r+p)\,\tilde{d}_i }{r \, k}\right]$ and an upper bound of $min\left( \left[ \frac{r+p}{r}\right] ^{(n-i)} a_{n}^{des} , 1\right)$. These bounds are proved in Ouzineb et al. (2014) (see Propositions 3.3 and 3.5 for more details). Note that $a_n=a_n^{des}$.
- $\tilde{d}_i, i=1 \ldots n$ is the long-term average number of parts pulled per unit time from stock $x_i$. It depends on the positions of the inspection stations. Let $e_j, j=1 \ldots m$ be the positions of the inspection stations, i.e.,
  3 $$\begin{aligned} \lambda _i = \left\{ \begin{array}{l l} 1 &{}\quad \text {if} \; i \in \{e_j,\, j=1 \ldots m\} \\ 0 & \quad{} \text {otherwise} \end{array}\right\} ,\quad i = 1 \ldots n-1. \end{aligned}$$
  Then we have
  4 $$\begin{aligned} \tilde{d}_i= \left\{ \begin{array}{l l} d(1+\beta )^n &{}\quad \text {if} \quad 1 \le i \le e_1 \\ d(1+\beta )^{n-e_1} &{} \quad \text {if} \quad e_1< i \le e_2 \\ .&{} \quad .\\ .&{} \quad .\\ d(1+\beta )^{n-e_{m-1}} &{} \quad \text {if} \quad e_{m-1}< i \le e_m \\ d(1+\beta )^{n-e_{m}} &{} \quad \text {if} \quad e_{m} < i \le n \end{array}\right\} , \end{aligned}$$
- and finally
  5 $$\begin{aligned}&T^{(i)}(a_i,a_{i-1},\lambda )= c_p\,\left( \frac{k\, \frac{(r(1-a_{i-1})+p)}{a_{i-1}}}{\sigma _i (k-\frac{\tilde{d}_i}{a_i})\,\frac{(r+p)}{a_{i-1}}}-\frac{k\ (1-a_i)}{\sigma _i (k-\frac{\tilde{d}_i}{a_i})} -\left[ \frac{1}{\sigma _i}-\frac{(1-a_i)\,\frac{(r+p)}{a_{i-1}}}{\sigma _i^2(k-\frac{\tilde{d}_i}{a_i})} \right] \right. \\&\quad \left. ln\left[ \frac{\frac{(r(1-a_{i-1})+p)}{a_{i-1}}\,\frac{\tilde{d}_i}{a_i}}{r \, (k-\frac{\tilde{d}_i}{a_i}) }-\frac{\sigma _i\, \frac{(r(1-a_{i-1})+p)}{a_{i-1}}\,\frac{\tilde{d}_i}{a_i}}{\frac{(r+p)}{a_{i-1}}\, r\,(1-a_i) }\right] \right) ,\quad i=1, \ldots , n-1, \end{aligned}$$
  6 $$\begin{aligned}&T_F(a^n_{des}, a_{n-1},\lambda ) = \\&\quad \frac{ \rho _n \, c_p\,\left( \frac{k\,(1- \exp (-\mu _n(1- \rho _n)\,z_n(a_{n}^{des}))}{1- \rho _n}-\frac{(r+p)}{a_{n-1}} z_n(a_{n}^{des})\exp (-\mu _n(1- \rho _n)\,z_n(a_{n}^{des})\right) }{\frac{(r+p)}{a_{n-1}} (1- \rho _n \,\exp (-\mu _n(1- \rho _n)\,z_n(a_{n}^{des})))} \end{aligned}$$
  with $$\sigma _i=\frac{ ({\frac{(r+p)}{a_{i-1}}})\, \frac{\tilde{d}_i}{a_i}-k\,r }{(k-\frac{\tilde{d}_i}{a_i})\,\frac{\tilde{d}_i}{a_i}}$$, $$\rho _n=\frac{r (k-\frac{\tilde{d}_n}{a_{n}^{des}})}{\frac{(r(1-a_{n-1})+p)}{a_{n-1}}\,\frac{\tilde{d}_n}{a_{n}^{des}}}$$, $$\mu _n=\frac{(r(1-a_{n-1})+p)}{a_{n-1} (k-\frac{\tilde{d}_n}{a_{n}^{des}})}$$, and $$z_n(a_{n}^{des}) = -\frac{\ln \left[ \frac{1}{\rho _n }\left( 1-\frac{(1- \rho _n)}{(1-a_{n}^{des})(\frac{(r+p)}{(r(1-a_{n-1})+p)})}\right) \right] }{\mu _n(1- \rho _n)}.$$

The problem can now be formulated as follows:

7 $$\begin{aligned}&\text{ minimize }&J(a, \lambda ) \end{aligned}$$

8 $$\begin{aligned}&\text{ subject } \text{ to }& \\& \sum _{i=1}^{n-1} \lambda _i =m \end{aligned}$$

9 $$\begin{aligned}&max\left[ \left( \frac{r}{(r+p)}\right) ^{i-1},\frac{(r+p)\,\tilde{d}_i}{r \, k}\right] \le a_{i} \le min\left( \left[ \frac{r+p}{r}\right] ^{(n-i)} a_{n}^{des} , 1\right) , \\&\,\forall i,\, 1 \le i \le n - 1 \end{aligned}$$

10 $$\begin{aligned}&\lambda _i \in \{0,1\},\;\;\forall i,\; 1 \le i \le n-1 \end{aligned}$$

11 $$\begin{aligned}&m \in \{1, 2, \ldots , n-1\}.\ \end{aligned}$$

We aim to find the optimal solution, i.e. the minimal average global cost system structure, $(a, \lambda , m)$ that minimizes *J* and satisfies the constraint (8) on the number of inspection stations, constraints (9) on buffers sizes bounds, and integrality constraints (10)–(11) on admissible values of $\lambda$ and *m*. The proposition below discusses the sensitivity of the production line to $c_I$. A numerical example is given in “Sensitivity analysis” section to illustrate this result.

### **Proposition 1**

*The total cost as a function of* $c_I$ *is a piecewise linear function. The slope of the line depends on the number of inspection stations* *m and their positions* $\lambda$.

### Proof

The minimum total storage and inspection cost is

$$\begin{aligned} J(a, \lambda ) = \sum _{i=1}^{n-1} T^{(i)}( a_i, a_{i-1},\lambda )+ T_F(a^n_{des}, a_{n-1},\lambda )+ c_I\,\sum _{i=1}^{n} \lambda _i \tilde{d}_i. \end{aligned}$$

The optimal total storage and inspection cost as a function of $c_I$ is

12 $$\begin{aligned} J(a^*, \lambda ^*)= & {} \sum _{i=1}^{n-1} T^{(i)}( a_i^*, a_{i-1}^*,\lambda ^*)+ T_F(a^n_{des}, a_{n-1}^*,\lambda ^*)+ c_I\,\sum _{i=1}^{n} \lambda _i^* \tilde{d}_i^* \\= & {}\, Constant(c_I)+ c_I\,\sum _{i=1}^{n} \lambda _i^* \tilde{d}_i^* \end{aligned}$$

where $(a^*, \lambda ^*)= arg min (J(a, \lambda ))$. Observe that $\sum _{i=1}^{n-1} T^{(i)}( a_i^*, a_{i-1}^*,\lambda ^*)+ T_F(a^n_{des}, a_{n-1}^*,\lambda ^*)$ does not depend on the value $c_I$, so it is constant on $c_I$ and referred to as $Constant(c_I)$. Thus, we can write Eq. (12) as follows:

13 $$\begin{aligned} J(a^*, \lambda ^*)= Constant(c_I)+ c_I\,\left( \sum _{i=1}^{m} d(1+\beta )^{n-e_{i}} \, + d(1+\beta )^{n}\right) . \end{aligned}$$

Hence, the total cost as a function of $c_I$ is a piecewise linear function; its slope depends only on *m* and the positions of the inspection stations. $\square$

The cost function *J* is likely to be convex as conjectured by Conjecture 1 and confirmed by the numerical results in “Characteristics of the production line” section. The convexity of the cost function is used to reduce the number of iterations. Actually, the local minimum of a convex function is also a global minimum, and there are many specialized methods and mathematical tools for optimizing convex functions.

### Conjecture 1

- The minimal total cost *J* is a convex function of $\lambda$.
- The minimal total cost *J* is a convex function of the number of inspection stations *m*.
- If we divide the production line into two parts with the same number of machines and we fix the number of stations in the first part, the minimal total cost *J* is a convex function of the number of internal stations in the second part.

## Methods

In Ouzineb et al. (2013, 2014), authors present an exact search methd (ESM) for the problem studied in this paper. For a given location of inspection stations, they show that the optimizing of the buffers’ sizes with ESM can be quickly done in polynomial time. Actually, the bottleneck of ESM is the number of possible locations. This number could be reduced by using the property of convexity (Conjecture 1) but nevertheless, it would stay very large. The innovation in this paper is to smartly select potential locations using an evolutionary algorithm combined with tabu search. We summarize ESM in “Exact search method (ESM)” section and then present our new approach in “Multilevel hybrid heuristic (MHH)” section.

### Exact search method (ESM)

For a fixed $\lambda$, Ouzineb et al. (2013, 2014) shows that the term $T^{(i)}(a_i, a_{i-1},\lambda )$, and thus the objective function itself, is separable by the variables $a_i$. ESM reformulates then the problem defined by (7)–(11), for a given $\lambda$, as a shortest path problem defined on a network (described below). It uses a standard shortest path algorithm to find the optimal buffers’ sizes. For this, we discretize the continuous variables $a_i$ to take discrete values 1, 2, ..., 100%. For a fixed $\lambda$, the value of *m* is determined straightforwardly. So, the constraints (8), (10), and (11) are satisfied. They do not depend on *a* and consequently could be omitted.

**Fig. 2:**
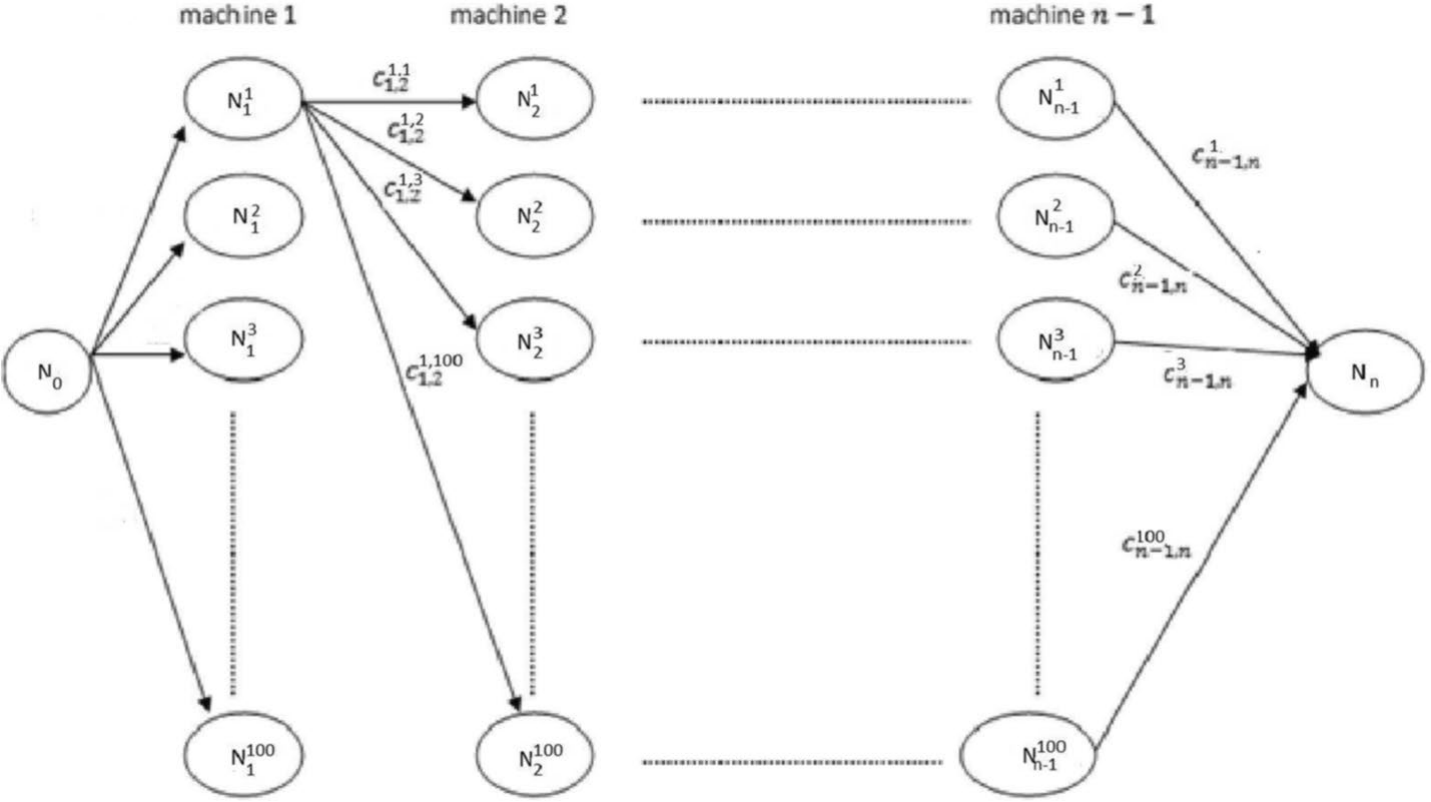
Network flow problem

Consider the connected network *G*(*N*, *A*) consisting of a set of nodes *N* and a set of arcs *A*, as depicted in Fig. 2. With each machine we associate a set of nodes representing all the possible buffers’ sizes, which range from 1 to 100%. The set *N* consists of $100 \times (n-1)$ nodes (representing the $n-1$ machines) plus two additional nodes $N_0$ and $N_n$ that are adjacent to the nodes representing the first and last machines. The set *A* consists of $100^2 \times (n-2)+200$ arcs weighted as follows:

- $N_0$ is connected to the nodes $N^j_1$, $j = 1,\dots ,100$ representing machine 1. The weight of each arc $(N_0,N^j_1)$ is null. $N_n$ is connected to the nodes $N^j_{n-1}$, $j = 1,\dots ,100$ representing machine $n-1$. The weight of each arc $(N^j_{n-1},N_n)$ is $c^{j}_{n-1,n}=T_{F} (a^{des}_n, j, \lambda )+c_I \times \tilde{d}_n$, $1\le j\le 100$.
- Each node $N^j_i$ representing machine *i* is connected to each node $N^k_{i+1}$ representing machine $i+1$ by an arc $(N^j_i,N^k_{i+1})$ that is weighted $c^{jk}_{i,i+1}=T^{(i)} (k,j,\lambda )+ c_I\times \lambda _i\times \tilde{d}_i$, $i = 1,\dots ,n-2$ and $1\le j,k \le 100$.

Nodes, and all incident arcs, corresponding to values that are not in the intervals imposed by constraints (9) are removed from the network. Algorithm 1 presents ESM. It finds optimal solutions but takes hours for instances with 20 machines. It is unable to solve some larger instances with 30 machines in one month!

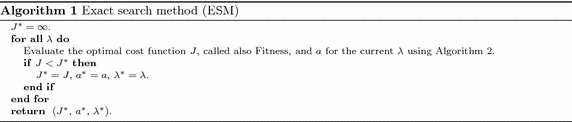

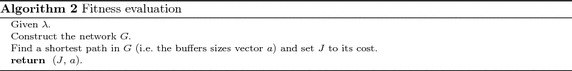





### Multilevel hybrid heuristic (MHH)

Our approach combines ideas from ESM and metaheuristic techniques. The choice of a search space and a neighborhood structure is a critical step in metaheuristic design (see Gendreau 2002). Our search space is the space of all possible line structures, i.e. the vectors (*a*, $\lambda$, *m*) that are feasible solutions to the system (8)–(11). As discussed above, the most determinant element of these vectors is $\lambda$ because if we know $\lambda$, we can compute *m* in a straightforward manner and *a* in a polynomial time. Consequently, our search space *S* in this section is composed of all possible $\lambda$ values. We reduce it by: (1) partitioning this space into a set of disjoint subspaces as explained in “Partitioning the search space” section; (2) applying a hybrid heuristic to selected subspaces in order to find potential solutions; we use Algorithm 2 above to evaluate the fitness of each selected $\lambda$ (in a subspace) and to find the optimal buffers’ sizes *a*. See “Hybrid heuristic” section for more details. The multilevel hybrid heuristic pseudocode is then given in “MHH pseudocode” section. This approach finds, as reported in the numerical results, near-optimal solutions in a fraction of the time required by ESM.

#### Partitioning the search space

We refer hereunder to $\lambda$ as a solution vector or simply solution. Each solution vector is assigned an address, also called a *level*, as explained below.

##### **Definition 1**

We divide the solution vector $\lambda$ into two equal parts and create its address *r* as follows: $r = Address(\lambda )= \sum _{l=1}^{\lfloor n/2 \rfloor }\lambda _l$. A search subspace of address *r*, denoted $S_r$, is defined to be the set of solutions that have the same address, i.e. equal to *r*.

The lower bound of *r* is 0 and its upper bound is *m*. It is clear that $(S_r)_{1\le r\le m}$ is a partition of the space *S*, i.e., $S_{r_1} \bigcap S_{r_2} =\emptyset \;\forall \, r_1\ne r_2$ and $S=\bigcup _{r=0}^{m} S_r$.

##### Example

Consider a system with $n=20$ machines and $m=5$ inspection stations: 2 stations in the first part and 3 in the second part:

$$\begin{aligned} {\varvec{\lambda }}=\left[ (0,0,1,0,0,0,0,1,0,0),({\mathbf{0, 0, 1, 0, 0, 1, 0, 0, 0, 1 }})\right] . \end{aligned}$$

From the above definition, $address({\varvec{\lambda }})= 2$. So, $\lambda \in S_2$.

#### Hybrid heuristic

**Fig. 3:**
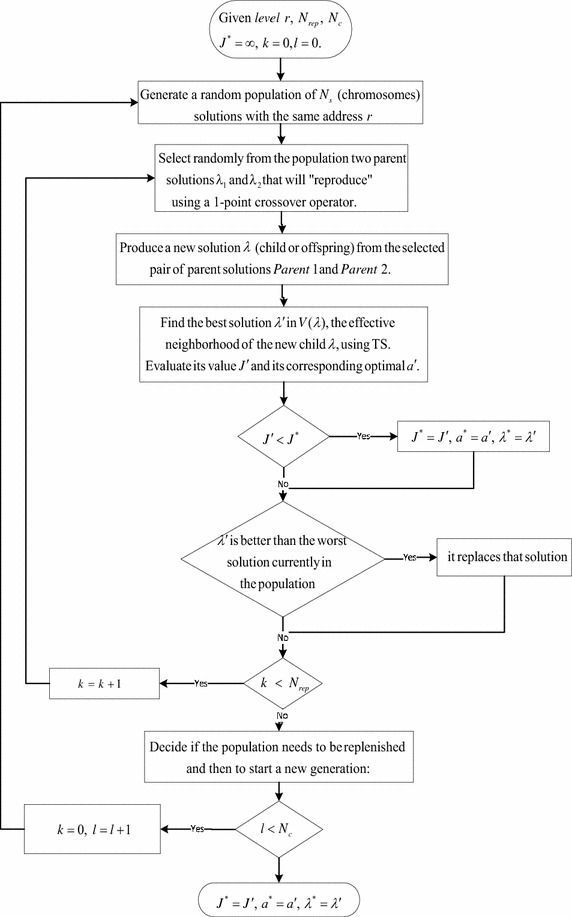
Hybrid heuristic algorithm

The goal here is to locate promising regions in a given subspace $S_r$. Our hybrid optimization is based on a combination of a GA, which provides diversification, and TS, which provides intensification. The hybrid heuristic pseudocode is presented in Algorithm 3 and a flowchart is given in Fig. 3. $N_{rep}$ and $N_c$ are GA tuned by experimentation. The main steps are explained in the paragraphs that follow.

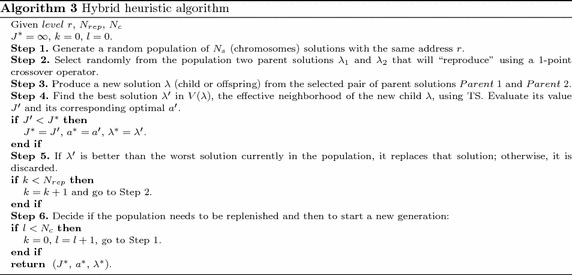



 In Step 3, we use a 1-point crossover operator, which, as illustrated in Table 1, creates a child string ${\varvec{\lambda }}$ for a given pair of parent strings ${\varvec{\lambda }}_1$ and ${\varvec{\lambda }}_2$ by:

1. Copying the string elements belonging to the first part from ${\varvec{\lambda }}_1$;
2. Copying the rest of the string elements from ${\varvec{\lambda }}_2$.

**Table 1:** Example of 1-point crossover operator

Parent string ${\varvec{\lambda }}_1$	0	0	0	1	0	0	1	0	0	0	0	1	0	0	0	0	0	1	0	1
Parent string ${\varvec{\lambda }}_2$	*0*	*0*	*0*	*0*	*1*	*0*	*1*	*0*	*0*	*0*	*0*	*1*	*1*	*0*	*0*	*0*	*0*	*0*	*0*	*1*
Child string	0	0	0	1	0	0	1	0	0	0	*0*	*1*	*1*	*0*	*0*	*0*	*0*	*0*	*0*	*1*

Italic values indictate the chromosome of Parent ${\varvec{\lambda }}_2$

Observe that the child has the same address as its parents.

In Step 4, we use TS, in which we iteratively move from the current solution $\lambda$ to a new solution in its neighborhood *Neighborhood*(${\varvec{\lambda }}$) until some stopping criterion has been satisfied. For a given $\lambda$, we define Neighborhood(${\varvec{\lambda }}$) = {solutions (inspection stations configurations) obtained by applying a single move to ${\varvec{\lambda }}$}. Moving to a new solution involves changing the positions of two inspection stations; the number of stations does not change. Note that we stay in the same level when we move to a neighbor solution. We use Algorithm 2 to evaluate the fitness of each new solution, and we use theses fitness values to compare different solutions.

TS uses a memory structure: a potential solution is declared *tabu* so that the algorithm does not visit it again; this prevents cycling. At each iteration, we select the best solution ${{\varvec{\lambda '}}}$ in a subspace V(${\varvec{\lambda }}$) $\subset$ Neighborhood(${\varvec{\lambda }}$) as a tabu solution. The subspace V(${\varvec{\lambda }}$), called the effective neighborhood, is obtained by eliminating the tabu solutions from Neighborhood(${\varvec{\lambda }}$). The tabu solutions are stored in a short-term memory, called a tabu list, which contains the solutions that have been visited in the recent past. We use a variable-size tabu list (tabu tenure) because this is generally more efficient (Gendreau 2002; Ouzineb et al. 2008). Finally, our stopping criterion is defined by the maximum number of local iterations (*mnli*), i.e. the number of visited neighbors that do not improve the best-known solution. The value of *mnli* is determined by the experimentation.

#### MHH pseudocode

**Fig. 4:**
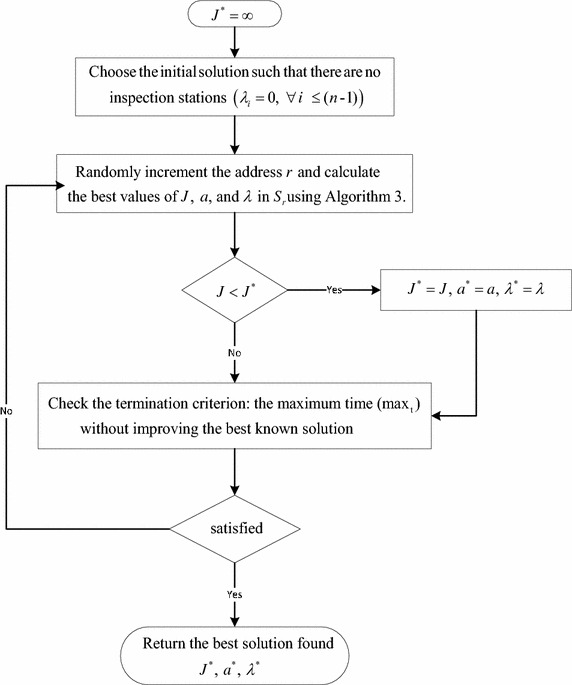
Multilevel hybrid heuristic algorithm

In MHH, at each iteration we move from the current level to a new level. We divide the search space into a set of disjoint subspaces (levels) using the partitioning technique of “Partitioning the search space” section and then apply the heuristic presented in “Hybrid heuristic” section to each selected subspace. Algorithm 4 presents the proposed pseudo code and Fig. 4 the corresponding flowchart.

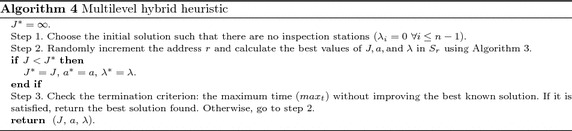



## Numerical results

We use four optimization problems (benchmarks) to test our algorithms: I10 has 10 machines, I20 has 20 machines, I30 has 30 machines and I40 has 40 machines. To get an insight into the algorithms behavior, we fixed the value of *m* in the model (7)–(11). The optimal objective value can be obtained equivalently by varying *m* between $1,\ldots , n-1$, sloving the resulting models and take the minimum possible. We implemented MHH and ESM in $C^{++}$ under the same conditions (computer architecture, operating system, etc). The tests were performed on an Intel Core i7 at 2.8 GHz with 8 GB of RAM, running Linux. Table 2 shows the parameters we used.

**Table 2:** Parameters

	*n*	*p*	*r*	*k*	$\beta$	*d*	$c_p$	$c_I$	$a_n^{des}$	$max_t (s)$	$N_{c}$	$N_{rep}$	*mnli*
10 machines	10	0.2	0.9	4	0.1	1	1	2	0.95	10	2	3	5
20 machines	20	0.2	0.9	9	0.1	1	0.1	0.2	0.95	600	2	5	5
30 machines	30	0.2	0.9	22	0.1	1	0.1	0.2	0.95	3600	2	5	5
40 machines	40	0.2	0.9	9	0.05	1	0.1	0.2	0.95	3600	2	2	5

### Comparing MHH to ESM

ESM can find the optimal solutions for I10 and I20. We compare the MHH solutions to the optimal solutions to evaluate the performance of MHH. ESM is unable to solve I30 and I40, which has been newly proposed in this paper, for large *m*. The reduction percentage in terms of CPU time (RPCPU) is defined as follows:

14 $$\begin{aligned} \text{ RPCPU }=100\times & {} \frac{(\text{ESM } \text{ Time }-\text{ MHH } \text{ Time) }}{\text{ ESM } \text{ Time }}\%.\\ \end{aligned}$$

For I10, MHH and ESM find the same optimal solutions in comparable times. Tables 3 and 4 give the results for I20 and I30, with $m=1, \ldots , 10$. The first column indicates the number *m* of inspection stations. The MHH cost is given in the second column; if the solution is optimal the entry is in italics. MHH finds optimal solutions for all the instances solved by ESM. The column MHH/Best gives the time to find the best solution, and the column MHH gives the total computational time. ESM takes hours on I20 for $m \ge 5$. MHH outperforms ESM in terms of the computational time; for I20, it finds optimal solutions in a fraction of ESM time. We also report results for a “pure” genetic algorithm (GA) which is obtained by modifying Algorithm 3 as follows:

- Remove Step 4.
- Replace Step 5 by: If the new solution is better than the worst solution currently in the population, it replaces that solution; otherwise, it is discarded.

GA is coded in the same technology and runs on the same machine as MHH and ESM. MHH results are signifcantly better in terms of quality and CPU time than GA. For larger instances, we omit GA and present results of MHH only.

**Table 3:** Results for I20

*m*	MHH cost	GA cost	Running time (s)				
			*ESM*	*MHH/Best*	*MHH*	*RPCPU*	GA
1	*8*.*3125*	9.23149	15.3	34.3	177.6	−91.39	457.0
2	*6*.*2645*	6.90041	105.7	77.4	440.5	−76.01	983.0
3	*5*.*9802*	6.28064	421.7	422.0	1011.9	−58.33	1524.4
4	*6*.*0663*	6.32419	1601.2	591.7	1191.7	25.58	1364.0
5	*6*.*2772*	6.51936	4743.3	571.2	879.4	81.46	1402.9
6	*6*.*5230*	6.76087	11,108.7	631.4	992.0	91.07	1649.9
7	*6*.*7959*	7.02709	20,613.0	635.2	1108.9	94.62	1710.7
8	*7*.*0965*	7.37334	29,790.2	960.7	1560.7	94.76	1255.8
9	*7*.*4298*	7.67294	35,764.9	476.5	1076.5	96.99	1176.9
10	*7*.*7947*	8.05445	35,767.6	109.0	709.0	98.02	977.7

Italic values indictate optimal costs

**Table 4:** Results for I30

*m*	MHH cost	Running time (s)			
		*ESM*	*MHH/Best*	*MHH*	*RPCPU*
1	*40.7297*	31.2	109.6	829.3	−96.24
2	*22.9847*	389.4	1186.7	1554.6	−74.95
3	*20.0887*	3524.2	3779.7	3926.9	−10.25
4	*19.0494*	18,769.2	4470.1	5671.9	69.78
5	*18.9133*	115,432.0	6584.2	8065.0	93.01
6	*19.1256*	421,630.0	10,034.2	13,634.2	96.77
7	19.4257	–	10,713.9	14,313.9	–
8	19.7315	–	12,300.6	15,900.6	–
9	20.1073	–	10,549.2	14,149.2	–
10	20.3278	–	11,128.0	14,728.0	–

Italic values indictate optimal costs

For I30, MHH finds “optimal” solutions in a reasonable time. ESM cannot solve the instances with $m \ge 7$. For example, for $m=7$, ESM did not converge after a month of running time. We believe that the MHH solution is optimal or near-optimal for $m = 7$ and beyond, but we cannot confirm this. Large problems require fast heuristics such as MHH that provide good results in an acceptable time.

Table 5 presents results for I40 which consists of very large instances. In this table, we report the cost values for both MHH and ESM because they are slightly different for $m = 2$ and $m = 3$. We did not compute *RPCPU* for the same reason. For I40, MHH finds good solutions in a reasonable time while ESM takes too much time for $m = 3$ and cannot solve the instances with $m \ge 4$; ESM did not converge after many hours of running time. The MHH solution is optimal or near-optimal for $m = 3$ and below. We think that the optimal solution is obtained when $m = 5$ despite the fact that we cannot solve it by the exact method.

**Table 5:** Results for I40

*m*	ESM cost	MHH cost	Running time (s)		
			ESM	*MHH/Best*	*MHH*
1	*25.2938*	*25.2938*	109,147	194.46	387.83
2	*16.0119*	16.1078	1105,72	1008.23	1013.94
3	*13.3200*	13.3208	13,200,38	2336.34	2623.08
4		12.5685		3932.31	5827.07
5		12.3450		6092.55	6920.45
6		12.5043		5266.51	11,511.62
7		12.6973		10,604.86	16,425.48
8		12.8781		18,929.32	23,346.94
9		13.1145		12,666.15	25,374.23
10		13.3623		15,501.52	31,699.22

Italic values indictate optimal costs

Table 6 shows the results for I20 when the computational time is limited to one hour. Both methods find optimal solutions for $m \le 4$; MHH finds better solutions for $m \ge 5$.

**Table 6:** MHH and ESM results for I20 with computational time limited to one hour

*m*	*MHH cost*	*ESM cost*
1	*8.3125*	*8.3125*
2	*6.2645*	*6.2645*
3	*5.9802*	*5.9802*
4	*6.0663*	*6.0663*
5	*6.2772*	6.4972
6	*6.5230*	7.0839
7	*6.7959*	7.4549
8	*7.0965*	7.8745
9	*7.4298*	8.4677
10	*7.7947*	8.8909

Italic values indictate optimal costs

### Characteristics of the production line

**Fig. 5:**
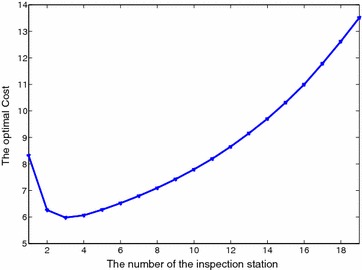
Optimal cost as a function of $m = 1,2, \ldots , 19$ for I20

Figure 5 shows the optimal cost as a function of (the number of internal inspection stations) $m = 1,2, \ldots , 19$. The cost is a convex function of *m*. The optimal number of internal inspection stations is $m=3$, and the optimal positions are $\lambda _2=1$, $\lambda _7=1$, and $\lambda _{18}=1$. The results obtained by MHH and ESM are identical.

**Fig. 6:**
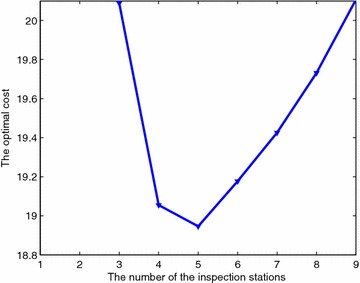
Optimal cost as a function of the number of inspection stations for I30

Figure 6 shows the best cost found by MHH as a function of $m = 1, 2, \ldots ,19$. The cost is a convex function of *m*. The optimal value for *m* is $m=5$, and the optimal positions are $\lambda _1=1$, $\lambda _3=1$, $\lambda _7=1$, $\lambda _{15}=1$, and $\lambda _{29}=1$. These results support Conjecture 1. Convexity can play an important role in reducing the solution time. Using convexity, we can conjecture that even I30 is solved optimally by ESM and MHH because the cost starts increasing for $m \ge 6$.

**Fig. 7:**
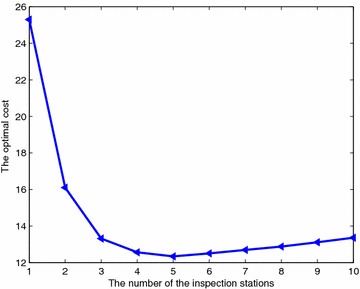
Optimal cost as a function of the number of inspection stations for I40

Figure 7 shows the best cost found by MHH as a function of $m = 1, 2, \ldots ,10$ for I40. The cost is a convex function of *m*. The optimal value for *m* is $m=5$.

### Sensitivity analysis

We now study the sensitivity of the optimal cost and number of inspection stations to the parameters of the model. We study particularily $\beta$ and $c_I$ because they directly affect quality. Table 7 gives the parameters used for this study; we investigate I20.

**Table 7:** Parameters

	*n*	*p*	*r*	*k*	*d*	$c_p$	$a_n^{des}$
20 machines	20	0.2	0.9	9	1	0.1	0.95

#### Inspection cost $c_I$

**Fig. 8:**
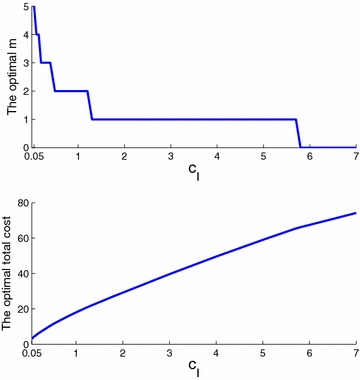
Optimal number of inspection stations and cost as a function of $c_I$

We begin by varying $c_I$, the inspection cost per time unit and per part (for a fixed $\beta =0.1$). The upper plot in Fig. 8 shows that for values of $c_I$ close to $c_p$ (the storage cost per time unit and per part), the optimal number of stations is high, while for high values of $c_I$, the optimal number decreases until it reaches zero. This is because the system encourages storage when it is profitable to do so, and it reduces nonconforming parts by imposing more inspection stations when that option is better.

When $c_I$ increases, by Proposition 1, $\left( \sum _{i=1}^{m} d(1+\beta )^{n-e_{i}} \right)$ must decrease (*m* or $(n-e_{i})$ decreases). The stations will thus be closer to the end of the line. When a station is placed after machine $n-1$, any increase in $c_I$ will make it unnecessary, so the optimal number of stations will be reduced by 1 (see Table 8).

**Table 8:** Optimal location of inspection stations

$c_I$	0.2	0.3	0.5	0.6	0.8	1.2	1.3	2	2.7	3.3	3.9	4.5	5.2	5.7
Optimal *m*	3	3	2	2	2	2	1	1	1	1	1	1	1	1
Optimal positions	2; 7; 18	2; 7; 19	3; 11	3; 13	3; 14	4; 19	5	6	7	8	9	10	11	11

**Fig. 9:**
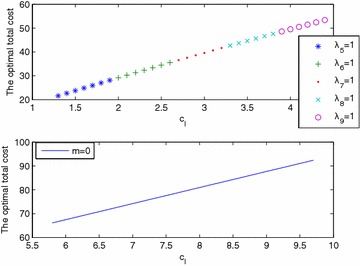
Optimal total cost as a function of $c_I$ ($m^*=1$ and $m^*=0$)

Figure 9 shows the cases $m=1$ (upper plot) and $m=0$ (lower plot). For $m=1$ and for each station position, the total cost as a function of $c_I$ is linear. For $m=0$, the total cost as a function of $c_I$ is linear.

#### Fraction $\beta$ of nonconforming to conforming parts

**Fig. 10:**
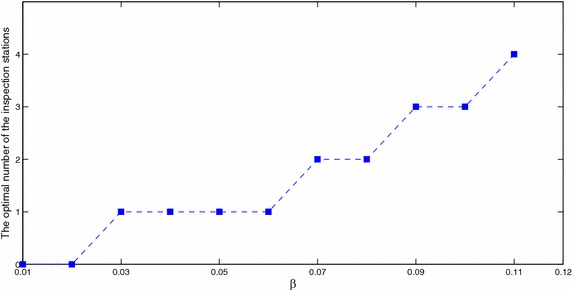
Optimal number of inspection stations as a function of $\beta$

**Fig. 11:**
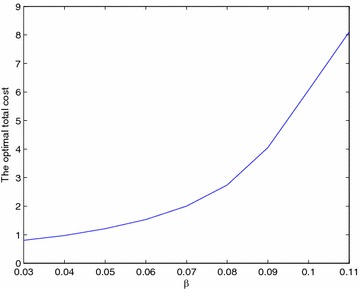
Optimal total cost as a function of $\beta$

Figure 10 and Table 9 show the behavior of the line as a function of $\beta$. As $\beta$ increases, the quantity of nonconforming parts increases. If we do not eliminate the nonconformity from the system, the machines will work harder and the stocks will fill faster, and the line will not be able to meet the final demand. Here the cost varies exponentially (Fig. 11).

**Table 9:** Optimal station locations

$\beta$	0.03	0.04	0.05	0.06	0.07	0.08	0.09	0.1	0.11
Optimal *m*	1	1	1	1	2	2	3	3	4
Optimal positions	18	18	18	16	8; 18	6; 18	4; 10; 19	2; 7; 18	2; 4; 8; 18

## Conclusions and future work

We proposed an efficient hybrid approach, based on ideas from an exact method and metaheuristics, for the buffer sizing problem in unreliable homogeneous production lines with several inspection stations. This is a difficult mixed integer nonlinear program. Our approach combines a genetic algorithm and tabu search to identify profitable configurations (locations of inspection stations). For these locations, we use an exact approach to decide the optimal buffers’ sizes. Our final goal is to find an optimal or near-optimal design as rapidly as possible. This hybrid approach provides a balance between diversification and intensification and works well on homogeneous production lines with up to 40 machines. MHH is significantly faster than ESM and produces solutions that are equally good.

Future research should focus on developing an optimization method for more realistic nonhomogeneous production lines and solving it by combining simulation and optimization. The model developed here could be used as an approximation model for more complex real life design problems. Fast optimization techniques, like the one proposed in this paper, would help in rapidly selecting potential scenarios/configurations for a more realistic simulation, and hence reducing the solution time without losing solution applicability in real life context.
